# Efficacy of second-line chemotherapy in patients with pulmonary large cell neuroendocrine carcinoma

**DOI:** 10.1038/s41598-024-58327-w

**Published:** 2024-04-01

**Authors:** Yuko Iida, Kazushige Wakuda, Hirotsugu Kenmotsu, Kosei Doshita, Hiroaki Kodama, Naoya Nishioka, Eriko Miyawaki, Taichi Miyawaki, Nobuaki Mamesaya, Haruki Kobayashi, Shota Omori, Ryo Ko, Akira Ono, Tateaki Naito, Haruyasu Murakami, Takashi Sugino, Yasuhiro Gon, Toshiaki Takahashi

**Affiliations:** 1 Division of Thoracic Oncology, Shizuoka Cancer Center, Shizuoka, Japan; 2 Division of Pathology, Shizuoka Cancer Center, Shizuoka, Japan; 3 Division of Respiratory Medicine, Department of Internal Medicine, Nihon University School of Medicine, Tokyo, Japan; 4 Respiratory Medicine and Infectious Diseases, Oita University Faculty of Medicine, Oita, Japan

**Keywords:** Cancer, Lung cancer

## Abstract

The efficacy of second-line chemotherapy in patients with pulmonary large cell neuroendocrine carcinoma (LCNEC) is unclear. This study aimed to evaluate the efficacy of second-line chemotherapy in patients with pulmonary LCNEC. We retrospectively reviewed patients with pulmonary LCNEC or possible LCNEC (pLCNEC) who received platinum-based chemotherapy as the first-line treatment. Among these patients, we evaluated the efficacy of second-line treatment by comparing patients with small cell lung cancer (SCLC group). Of the 61 patients with LCNEC or pLCNEC (LCNEC group) who received first-line chemotherapy, 39 patients were treated with second-line chemotherapy. Among the 39 patients, 61.5% received amrubicin monotherapy. The median progression-free survival (PFS) and overall survival (OS) in the LCNEC groups were 3.3 and 8.3 months, respectively. No significant differences in the PFS (hazard ratio [HR]: 0.924, 95% confidence interval [CI] 0.647–1.320; *P* = 0.664) and OS (HR: 0.926; 95% CI 0.648–1.321; *P* = 0.670) were observed between the LCNEC and SCLC groups. In patients treated with amrubicin, the PFS (*P* = 0.964) and OS (*P* = 0.544) were not different between both the groups. Second-line chemotherapy, including amrubicin, may be considered as a treatment option for patients with pulmonary LCNEC.

## Introduction

Lung neuroendocrine carcinoma is classified as large cell neuroendocrine carcinoma (LCNEC) and small cell lung cancer (SCLC) according to the classification by the World Health Organization. LCNEC, which accounts for 3% of resected lung cancers, is defined as a type of high-grade non-small cell carcinoma with neuroendocrine morphology and mitotic count of > 10 mitoses/2 mm^2^ expressing one or more neuroendocrine immunohistochemical markers^1^. LCNEC is mainly diagnosed in surgical specimens, and definitive diagnosis in a small biopsy is sometimes challenging since it cannot accurately evaluate the neuroendocrine morphology and/or immunohistochemical markers. Diagnosis suspected LCNEC in small biopsy specimens is defined as “non-small cell lung carcinoma, possible large cell neuroendocrine carcinoma”^2^. A standard chemotherapy regimen for patients with advanced LCNEC has not yet been established owing to the rarity of the tumor.

For patients with SCLC, a standard therapeutic strategy has been established for first- and second-line chemotherapy^3^. The efficacy of topotecan in patients with relapsed SCLC has been demonstrated^4–7^. Carboplatin and etoposide therapy were superior to topotecan in terms of progression-free survival (PFS) and overall response rate (ORR) in a randomized phase III trial for patients with sensitive relapsed SCLC^8^. In a phase III trial comparing amrubicin with topotecan, no significant difference in the overall survival (OS) was observed between the two groups. However, amrubicin significantly improved the OS in a subset of patients with refractory relapsed SCLC^9^. Some Japanese phase II trials have demonstrated the efficacy of amrubicin in patients with refractory relapsed SCLC^10–12^. Based on these results, amrubicin has been used as a standard treatment for patients with refractory relapsed SCLC in Japan^13^.

No randomized trials have evaluated the efficacy of first-line chemotherapy in this rare population of patients with LCNEC. SCLC-or non-small cell lung cancer (NSCLC)-based chemotherapy was reportedly effective as first-line chemotherapy for patients with LCNEC in a retrospective study^14–17^. Only two prospective single-arm phase II trials demonstrating the safety and efficacy of cisplatin plus etoposide or cisplatin plus irinotecan as first-line chemotherapy for patients with advanced or metastatic LCNEC have been reported^18,19^. The American Society of Clinical Oncology (ASCO) guidelines suggest the use of platinum plus etoposide chemotherapy for optimal efficacy^20^. Second-line chemotherapy for patients with pulmonary LCNEC has not been established since no prospective trials have evaluated subsequent chemotherapy in this population. Therefore, we conducted this retrospective study to investigate the efficacies of second-line chemotherapy in patients with advanced or metastatic pulmonary LCNEC.

## Methods

### Patients

From September 2004 to April 2021, patients with advanced or recurrent pulmonary LCNEC, including possible LCNEC (pLCNEC), and SCLC who received platinum doublet chemotherapy as first-line treatment at Shizuoka Cancer Center (Shizuoka, Japan) were retrospectively evaluated based on data from the medical records. To assess the efficacy of second-line chemotherapy for patients with LCNEC or pLCNEC, we compared patients with LCNEC or pLCNEC who received second-line chemotherapy (LCNEC group) to those with SCLC who received second-line chemotherapy (SCLC group). In this study, possible LCNEC was diagnosed as NSCLC where neuroendocrine morphology and/or marker expression is not definitive in scant or disrupted samples, based on the 2021 WHO Classification of Thoracic Tumors^2,21,22^. Second-line chemotherapy was defined as systemic chemotherapy following platinum doublet chemotherapy for advanced or recurrent LCNEC, including post-operative recurrence. The treatment-free interval (TFI) was defined as the period from the last first-line chemotherapy dose to the first relapse, and patients were divided into > 90 days and ≤ 90 days groups (Table 1). In patients who received postoperative adjuvant chemotherapy, adjuvant chemotherapy was considered first-line chemotherapy if recurrence occurred within 6 months of the last dose of adjuvant chemotherapy. In this study, the complication of interstitial lung disease (ILD) was defined as having interstitial lung abnormalities, which are a set of radiological abnormalities, such as ground glass opacities, subpleural reticulation, traction bronchiectasis, centrilobular nodules, honeycombing, and architectural distortion^23,24^. The distant metastasis at the start of second-line chemotherapy was defined as separate tumor nodule(s) in a contralateral lobe or tumor with pleural or pericardial nodule(s) or malignant pleural or pericardial effusion or extrathoracic metastasis, according to the 8th edition of the TNM classification^25^. This study was performed in accordance with the principles of the Declaration of Helsinki and all applicable requirements in Japan, was approved by institutional review boards at the Shizuoka Cancer Center (approval number: J2021-37). Because this was a retrospective study, informed consent was waived by institutional review boards of the Shizuoka Cancer Center. We provided the patients the opportunity to refuse this study enrollment, by using web page.

**Table 1:** Patient characteristics at the start of second-line chemotherapy.

	LCNEC	SCLC	*P* value
N	39	431	
Median age (range)	68 (55–83)	70 (43–87)	0.169
Sex, n (%)			
Female	7 (17.9)	73 (16.9)	0.826
Male	32 (82.1)	358 (83.1)	
Smoking status, n (%)			
Never	1 (2.6)	5 (1.2)	0.407
Ever smoker	38 (97.4)	426 (98.8)	
ECOG performance status, n (%)			
0	8 (20.5)	107 (24.8)	0.896
1	26 (66.7)	268 (62.2)	
2	5 (12.8)	52 (12.1)	
3	0 (0.0)	3 (0.7)	
4	0 (0.0)	1 (0.2)	
LCNEC	19		
pLCNEC	20		
Stage at initial diagnosis (8th TMN classification), n (%)			
1	4 (10.3)	7 (1.6)	0.012
2	2 (5.1)	17 (3.9)	
3	13 (33.3)	117 (27.1)	
4	20 (51.3)	290 (67.3)	
Treatment-free interval, n (%)			
> 90 days	13 (33.3)	178 (41.3)	0.396
≤ 90 days	26 (66.7)	253 (58.7)	
History of thoracic radiation therapy, n (%)			
No	31 (79.5)	343 (79.6)	1.000
Yes	8 (20.5)	88 (20.4)	
History of thoracic surgery, n (%)			
No	30 (76.9)	411 (95.4)	< 0.001
Yes	9 (23.1)	20 (4.6)	
Complication of ILD, n (%)			
No	37 (94.9)	338 (78.4)	0.012
Yes	2 (5.1)	93 (21.6)	
CNS metastasis at the start of second-line chemotherapy, n (%)			
No	23 (59.0)	211 (49.0)	0.395
Yes	14 (35.9)	173 (40.1)	
Not evaluated	2 (5.1)	47 (10.9)	
Distant metastasis at the start of second-line chemotherapy, n (%)			
No	3 (7.7)	53 (12.3)	0.604
Yes	36 (92.3)	378 (87.7)	
Median duration from the start of initial treatment to second-line chemotherapy (days) (range)	194 (21–1633)	199 (21–2373)	0.783
First-line chemotherapy, n (%)			
CDDP/CBDCA + ETP	20 (51.3)	328 (76.1)	
CDDP + CPT-11	12 (30.8)	70 (16.2)	
CBDCA + ETP + atezolizmab	0 (0.0)	11 (2.6)	
CDDP/CBDCA + ETP + durvalumab	0 (0.0)	4 (0.9)	
CBDCA + paclitaxel	6 (15.4)	0 (0.0)	
CBDCA + S-1	1 (2.6)	0 (0.0)	
Investigation drugs	0 (0.0)	18 (4.2)	
First-line chemotherapy response, n (%)			
CR	2 (5.1)	15 (3.5)	< 0.001
PR	16 (41.0)	365 (84.7)	
SD	11 (28.2)	32 (7.4)	
PD	8 (20.5)	12 (2.8)	
Not evaluate	2 (5.1)	7 (1.6)	

*CBDCA* carboplatin, *CDDP* cisplatin, *CNS*: central nervous system, *CPT-11* irinotecan, *ECOG* Eastern Cooperative Oncology Group, *ETP* etoposide, *ILD* interstitial lung disease, *LCNEC* large cell neuroendocrine carcinoma, *SCLC* small cell lung cancer.

### Evaluation

The best response to treatment was evaluated according to the response evaluation criteria in solid tumors (RECIST version 1.1)^26^. The evaluation of the treatment response using computed tomography was predominantly performed in every two courses of chemotherapy. Clinical evaluation of PFS following the start of second-line chemotherapy was performed based on the time of recurrence or death. OS was defined as the time from the date of second-line treatment to death.

### Statistical analysis

All the categorical variables were analyzed using Fisher's exact test, and continuous variables were evaluated using the Mann–Whitney *U* test. PFS and OS distributions were estimated using the Kaplan–Meier method. The log-rank test was used to compare the cumulative survival in each group. Hazard ratios (HRs) and confidence intervals (CIs) were estimated by Cox proportional hazards models. All the statistical analyses were performed using EZR (Saitama Medical Center, Jichi Medical University, Saitama, Japan), a graphical interface for R (The R Foundation for Statistical Computing, Vienna, Austria)^27^. All *P* values were reported as two-sided, and values < 0.05 were considered statistically significant.

## Results

### Patient characteristics

**Figure 1:**
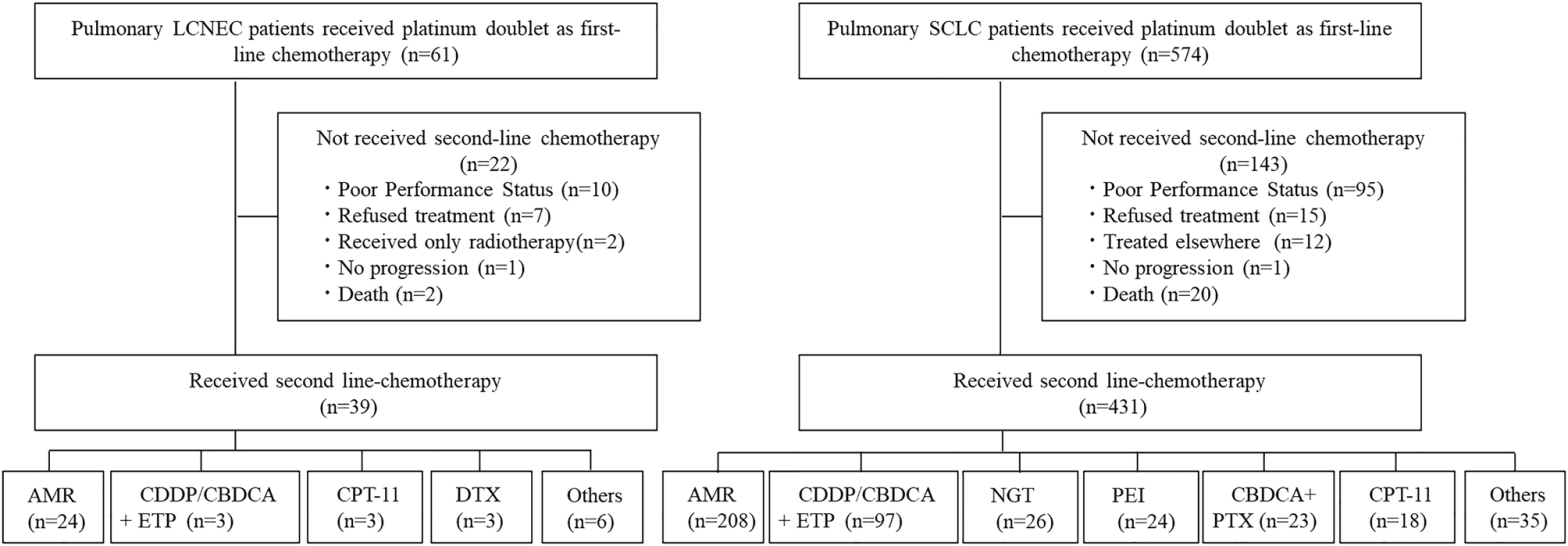
Patients with large cell neuroendocrine carcinoma (LCNEC) and small cell lung cancer (SCLC) enrolled in this study. *AMR* amrubicin, *CDDP* cisplatin, *CBDCA* carboplatin, *ETP* etoposide, *CPT-11* irinotecan, *DTX* docetaxel, *NGT* topotecan, *PEI* cisplatin, etoposide, and irinotecan, *PTX* paclitaxel.

Among the 61 patients with advanced or recurrent pulmonary LCNEC or pLCNEC who received platinum combined chemotherapy as first-line treatment, 39 patients received second-line chemotherapy. Twenty-two patients were not treated with second-line chemotherapy owing to poor performance status (PS) (n = 10), patient refusal (n = 7), received only radiotherapy for local recurrence (n = 2), no progression (n = 1), or death (n = 2) (Fig. 1). Among the 39 patients who received second line chemotherapy in the LCNEC group, the median age was 68 years and 82.1% were male. The patients with 97.4% had smoking history, and the Eastern Cooperative Oncology Group—PS 0, 1, and 2 was 20.5%, 66.7%, and 12.8%, respectively. Stages I, II, III, and IV at diagnosis were 10.3%, 5.1%, 33.3%, and 51.3%, respectively. TFI > 90 days was observed in 33.3% and ≤ 90 days was in 66.7% (Table 1). There were significant differences in stage at diagnosis, history of thoracic surgery, first-line chemotherapy response, and ILD complications between the LCNEC and SCLC groups.

### Efficacy of second-line chemotherapy in the LCNEC groups

The most frequently used regimen was amrubicin monotherapy (61.5%). Other regimens included platinum plus etoposide (7.7%), irinotecan monotherapy (7.7%), and docetaxel monotherapy (7.7%) (Table 2). In the LCNEC group, three patients achieved partial response (PR), and the overall response rate (ORR) was 7.7% (95% confidence interval [CI] 1.6–20.9%) (Table 3). The median PFS was 3.3 months (95%CI 1.4–4.9 months) and the median OS was 8.3 months (95% CI 4.5–11.7 months) in the LCNEC group. In the patients who received amrubicin monotherapy, the ORR was 8.3% (95% CI 1.0–27.0%) in the LCNEC group (Table 3). The median PFS and the median OS were 2.8 months (95% CI 1.2–4.9 months) and 8.3 months (95% CI 4.5–12.0 months), respectively in the LCNEC group. Between patients with LCNEC and pLCNEC, there were no significant differences in the ORR (5.3% and 10.0%, *P* = 0.359), PFS (median 1.5 months and 3.3 months, HR: 0.797, 95% CI 0.396–1.603, *P* = 0.525), and OS (median 8.3 months and 6.9 months, HR: 1.052, 95% CI 0.552–2.121, *P* = 0.886) (Supplementary Fig. S1).

**Table 2:** Second-line chemotherapy regimens.

Treatment	LCNEC (n = 39)	SCLC (n = 431)
	n (%)	n (%)
AMR	24 (61.5)	208 (48.3)
CDDP/CBDCA + ETP	3 (7.7)	97 (22.5)
CPT-11	3 (7.7)	18 (4.2)
DTX	3 (7.7)	0 (0.0)
NGT	1 (2.6)	26 (6.0)
PEI	1 (2.6)	24 (5.6)
CBDCA + PTX	1 (2.6)	23 (5.3)
PI	1 (2.6)	16 (3.7)
PTX	1 (2.6)	13 (3.0)
S-1	1 (2.6)	0 (0.0)
GEM	0 (0.0)	2 (0.5)
nab-PTX	0 (0.0)	1 (0.2)
CBDCA + nab-PTX	0 (0.0)	1 (0.2)
Investigation drugs	0 (0.0)	2 (0.5)

*AMR* amrubicin, *CDDP* cisplatin, *CBDCA* carboplatin, *ETP* etoposide, *CPT-11* irinotecan, *DTX* docetaxel, *GEM* gemcitabine, *NGT* topotecan, *PEI* cisplatin, etoposide, and irinotecan, *PI* cisplatin and irinotecan, *PTX* paclitaxel.

**Table 3:** Response to second-line chemotherapy.

	LCNEC	SCLC	*P* value
All			
Response, n (%)			
CR	0 (0.0)	5 (1.2)	0.002
PR	3 (7.7)	150 (34.8)	
SD	18 (46.2)	135 (31.3)	
PD	13 (33.3)	114 (26.5)	
Not evaluate	5 (12.8)	27 (6.3)	
Overall response rate (%)	7.7 (95% CI 1.6–20.9)	36.0 (95% CI 31.4–40.7)	< 0.001
Patients receiving amrubicin monotherapy			
Response, n (%)			
CR	0 (0.0)	1 (0.5)	0.002
PR	2 (8.3)	63 (30.3)	
SD	11 (45.8)	66 (31.7)	
PD	9 (37.5)	66 (31.7)	
Not evaluate	2 (8.3)	12 (5.8)	
Overall response rate (%)	8.3 (95% CI 1.0–27.0)	30.8 (95% CI 24.6–37.5)	0.029

*CR* complete response, *PD* progressive disease, *PR* partial response, *SD* stable disease, *CI* confidence interval.

### Second-line chemotherapy efficacy in the LCNEC groups compared to the SCLC groups

**Figure 2:**
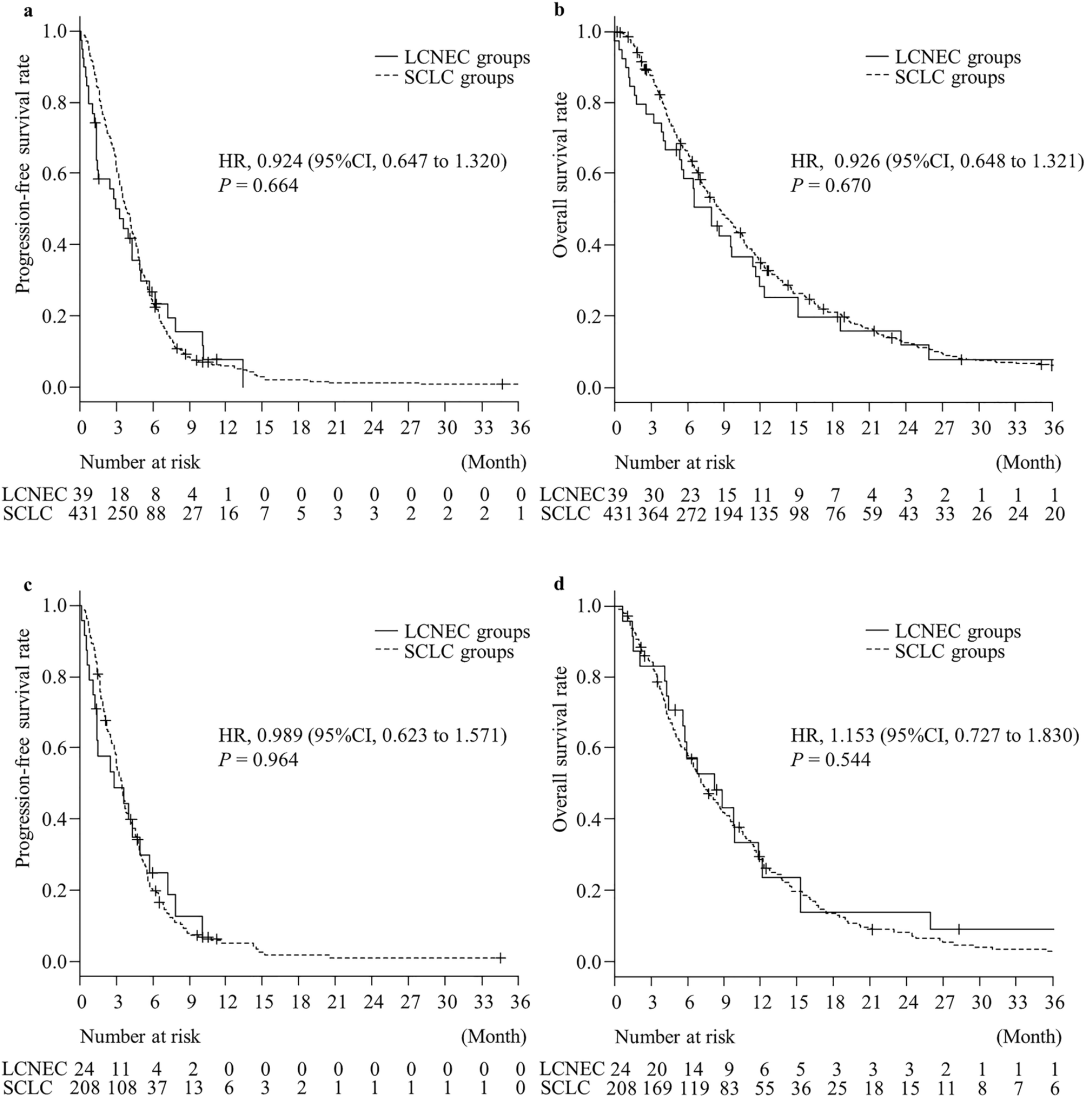
Kaplan–Meier survival curves for (**a**) progression free survival (PFS) and (**b**) overall survival (OS) between the large cell neuroendocrine carcinoma (LCNEC) and small cell lung cancer (SCLC) groups receiving second-line chemotherapy. Kaplan–Meier survival curves for (**c**) PFS and (**d**) OS for second-line chemotherapy between the LCNEC and SCLC groups receiving the amrubicin monotherapy. Survival curve of LCNEC (continuous line) and SCLC (dotted line line) are analyzed using the log-rank test.

There were no significant differences in the patient characteristics between the LCNEC and SCLC groups, except for the stage at diagnosis, history of thoracic surgery, and complications of ILD (Table 1). There were no significant differences in the number of patients receiving third-line chemotherapy between the two groups (*P* = 0.184). The ORR was significantly lower in the LCNEC than in the SCLC group (*P* < 0.001). There were no significant differences in the PFS (HR: 0.924, 95% CI 0.647–1.320, *P* = 0.664) and OS (HR: 0.926, 95% CI 0.648–1.321, *P* = 0.670) between the LCNEC and SCLC groups (Fig. 2A and B). In the subgroup of patients with TFI > 90, there were no significant differences in the PFS (HR: 1.336, 95% CI 0.681–2.624, *P* = 0.396) and OS (HR: 1.312, 95% CI 0.670–2.570, *P* = 0.427) between the LCNEC and SCLC groups. In patients with TFI ≤ 90, significant differences in the PFS (HR: 0.795, 95% CI 0.521–1.212, *P* = 0.284) and OS (HR: 0.792, 95% CI 0.519–1.209, *P* = 0.278) were not observed between both the groups. In patients who received amrubicin monotherapy, the ORR was also significantly lower in the LCNEC than in the SCLC group (*P* = 0.029) (Table 3). There were no significant differences in the PFS (HR: 0.989, 95% CI 0.623–1.571, *P* = 0.964) and OS (HR: 1.153, 95% CI 0.727–1.830, *P* = 0.544) between the LCNEC and SCLC groups (Figure 2C and D).

### The OS from the progression of first-line chemotherapy between the patients with LCNEC and pLCNEC who received and did not receive second-line chemotherapy

**Figure 3:**
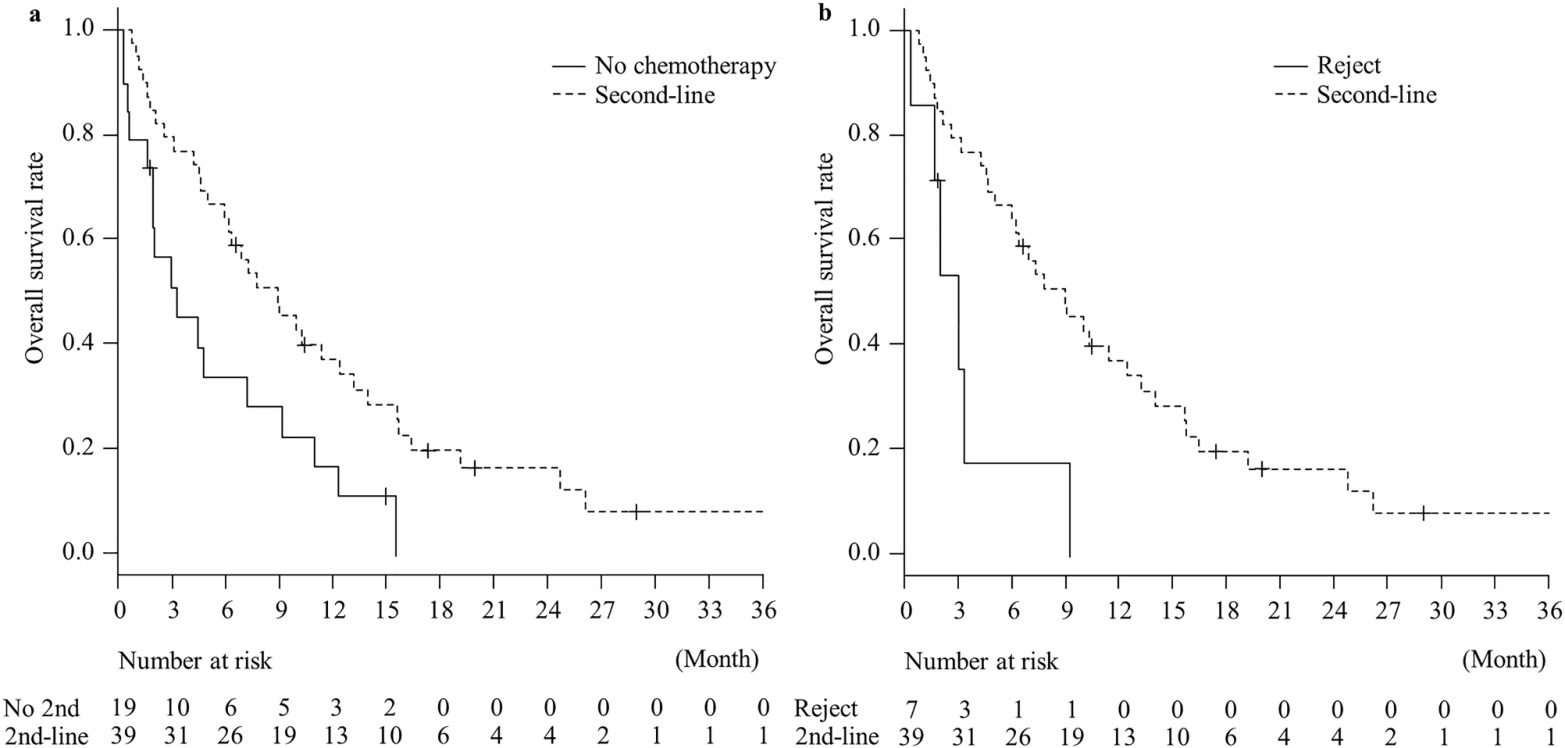
Kaplan–Meier survival curves for overall survival time from the progression of first-line chemotherapy between the patients who received second-line chemotherapy and those who did not. Survival curve of the patients who did not receive (No 2nd chemotherapy) (continuous line) and those who received second-line chemotherapy (2nd-line) (dotted line) are shown (**a**). Survival curve of the patients who did not receive second-line chemotherapy owing to patient refusal (Reject) (continuous line) and those received second-line chemotherapy (2nd-line) (dotted line line) are shown (**b**).

The median OS time from the progression of first-line chemotherapy in the patients with LCNEC / pLCNEC who received and did not receive second-line chemotherapy was 9.0 (95% CI: 5.1–12.5 months) and 3.4 (95% CI 1.7–7.3 months) months, respectively (Fig. 3A). Even in those who did not receive second-line chemotherapy due to patient refusal, the median OS from the progression of first-line chemotherapy was 3.1 months (95% CI 0.3–NA months) (Fig. 3B).

## Discussion

The present retrospective study demonstrated that the PFS and OS of patients with LCNEC were comparable to those of patients with SCLC receiving second-line chemotherapy. In the first-line treatment, the standard chemotherapy for patients with LCNEC is still controversial as to whether the SCLC- or NSCLC based regimen is more effective. Randomized controlled trials have not been performed, and both SCLC and NSCLC-based regimens have been shown to be effective for patients with LCNEC in some retrospective studies^14–17^. In two prospective phase II studies, the efficacy and tolerability of SCLC-based chemotherapy were demonstrated. The median PFS and median OS of patients treated with cisplatin plus etoposide were 5.2 and 7.7 months, respectively, in patients with LCNEC^18^. Moreover, Niho et al. reported that the ORR of cisplatin plus irinotecan was 46.7%, median PFS was 5.2 months, and median OS was 12.6 months in patients with LCNEC. In this report, according to the central pathological review, 73% were diagnosed with LCNEC, and 24% were diagnosed with SCLC. The authors showed that the ORR and OS in patients with LCNEC tended to be inferior to those in patients with SCLC^19^. According to the ASCO guidelines, platinum plus etoposide combination therapy may provide optimal efficacy for patients with LCNEC^20^.

A standard regimen of second-line chemotherapy does not exist for patients with LCNEC since no prospective study has evaluated the efficacy of second-line chemotherapy for this population. Two retrospective studies, including a small number of patients with LCNEC, evaluated amrubicin monotherapy as a second-line treatment. Kasahara et al. have reported an ORR of 11.1%, median PFS of 4.0 months, and median OS of 9.1 months for amrubicin monotherapy as second-line treatment in 18 patients with pulmonary LCNEC or high-grade non-small cell neuroendocrine carcinoma^28^. Yoshida et al. reported the efficacy of amrubicin in 18 previously treated patients with advanced pulmonary LCNEC; the ORR was 27.7%, median PFS was 3.1 months, and median OS was 5.1 months^29^. To our knowledge, our study is a relatively large retrospective study that evaluated the efficacy of second-line chemotherapy in patients with LCNEC/pLCNEC. Although LCNEC is not a common histological type of lung cancer, data on second-line treatments are still important in clinical settings.

The present study showed no significant differences in the PFS and OS between patients with LCNEC and SCLC; however, ORR was significantly lower in patients with LCNEC than those with SCLC in second-line chemotherapy. Shimada et al. have also reported that the ORR tended to be lower in patients with high-grade neuroendocrine carcinoma–probable LCNEC diagnosed in biopsy specimens compared to patients with SCLC (17% and 43%, respectively; *P* = 0.12)^30^. However, our report showed no significant differences in the PFS and OS between patients with LCNEC and SCLC. This result could be due to no difference in the rate of progressive disease between the LCNEC and SCLC groups (33.3% and 26.5%, respectively). Further, the OS from the progression of first-line chemotherapy tends to be longer in the patients who received second-line chemotherapy compared to those who did not receive second-line chemotherapy owing to the patient`s refusal. Based on these results, second-line chemotherapy for patients with LCNEC could be a treatment option since performing randomized controlled trials for second-line treatment in patients with LCNEC is challenging. Furthermore, we reported no significant difference in the PFS and OS in patients receiving amrubicin monotherapy between the LCNEC and SCLC groups. Our results suggest that amrubicin monotherapy is an option for second-line treatment in LCNEC.

This study has several limitations. First, the sample size was small in this single-center Japanese retrospective study. However, LCNEC is rare, and few reports exist on second-line chemotherapy in LCNEC, and this study included the largest number of patients with LCNEC/pLCNEC. Second, there were differences in initial stage, history of thoracic surgery, first-line chemotherapy response, and ILD complications between the LCNEC and SCLC groups. LCNEC is mainly diagnosed using surgical specimens, and definitive diagnosis in a small biopsy sample is sometimes difficult. A previous report showed that only 2.8% of all SCLC cases received surgical resection, and this population seemed to be rare as well as our study^31^. Third, the combination of other histological types in LCNEC/pLCNEC diagnosed using biopsy specimens cannot be completely ruled out. However, most cases of LCNEC/pLCNEC in advanced stage are diagnosed using biopsy specimens in a clinical setting. Furthermore, a previous report showed that there were no differences in the chemotherapeutic efficacy between LCNEC diagnosed using surgically resected specimens and pLCNEC diagnosed using small biopsy specimens^32^. Therefore, including biopsy specimens to examine the efficacy of second-line chemotherapy in patients with LCNEC is considered appropriate. Fourth, no patient in this study received immunotherapy or anti-angiogenesis therapy because these treatments had not been approved for second-line chemotherapy in Japan. Real world data of LCNEC patients receiving immune-checkpoint inhibitors seems necessary.

## Conclusions

This retrospective study showed no significant differences in the PFS and OS between patients with pulmonary LCNEC who received second-line chemotherapy and those with SCLC. Therefore, second-line chemotherapy including amrubicin may be considered as a treatment option for patients with pulmonary LCNEC.

### Supplementary Information

Supplementary Figure S1.

Supplementary Table 1.

## Data Availability

All data and material are available from the corresponding author on reasonable request.

## References

[1] Takei H (2002). Large cell neuroendocrine carcinoma of the lung: A clinicopathologic study of eighty-seven cases. J. Thorac. Cardiovasc. Surg..

[2] WHO Classification of Tumours Editorial Board (2021). WHO Classification of Tumours Thoracic Tumors.

[3] Zugazagoitia J, Paz-Ares L (2022). Extensive-stage small-cell lung cancer: First-line and second-line treatment options. J. Clin. Oncol..

[4] O’Brien MER (2006). Phase III trial comparing supportive care alone with supportive care with oral topotecan in patients with relapsed small-cell lung cancer. J. Clin. Oncol..

[5] Eckardt JR (2007). Phase III study of oral compared with intravenous topotecan as second-line therapy in small-cell lung cancer. J. Clin. Oncol..

[6] von Pawel J (1999). Topotecan versus cyclophosphamide, doxorubicin, and vincristine for the treatment of recurrent small-cell lung cancer. J. Clin. Oncol..

[7] Takeda K (2003). A phase II study of topotecan in patients with relapsed small-cell lung cancer. Clin. Lung Cancer.

[8] Baize N (2020). Carboplatin plus etoposide versus topotecan as second-line treatment for patients with sensitive relapsed small-cell lung cancer: An open-label, multicentre, randomised, phase 3 trial. Lancet Oncol..

[9] von Pawel J (2014). Randomized phase III trial of amrubicin versus topotecan as second-line treatment for patients with small-cell lung cancer. J. Clin. Oncol..

[10] Onoda S (2006). Phase II trial of amrubicin for treatment of refractory or relapsed small-cell lung cancer: Thoracic Oncology Research Group Study 0301. J. Clin. Oncol..

[11] Inoue A (2008). Randomized phase II trial comparing amrubicin with topotecan in patients with previously treated small-cell lung cancer: North Japan Lung Cancer Study Group Trial 0402. J. Clin. Oncol..

[12] Murakami H (2014). A single-arm confirmatory study of amrubicin therapy in patients with refractory small-cell lung cancer: Japan Clinical Oncology Group Study (JCOG0901). Lung Cancer.

[13] Horita N (2016). Amrubicin for relapsed small-cell lung cancer: A systematic review and meta-analysis of 803 patients. Sci. Rep..

[14] Rossi G (2005). Role of chemotherapy and the receptor tyrosine kinases KIT, PDGFRalpha, PDGFRbeta, and Met in large-cell neuroendocrine carcinoma of the lung. J. Clin. Oncol..

[15] Sun JM (2012). Chemotherapy for pulmonary large cell neuroendocrine carcinoma: similar to that for small cell lung cancer or non-small cell lung cancer?. Lung Cancer.

[16] Naidoo J (2016). Large cell neuroendocrine carcinoma of the lung: Clinico-pathologic features, treatment, and outcomes. Clin. Lung Cancer.

[17] Derks JL (2017). Chemotherapy for pulmonary large cell neuroendocrine carcinomas: Does the regimen matter?. Eur. Respir. J..

[18] Le Treut J (2013). Multicentre phase II study of cisplatin-etoposide chemotherapy for advanced large-cell neuroendocrine lung carcinoma: The GFPC 0302 study. Ann. Oncol..

[19] Niho S (2013). Combination chemotherapy with irinotecan and cisplatin for large-cell neuroendocrine carcinoma of the lung: A multicenter phase II study. J. Thorac. Oncol..

[20] Masters GA (2015). Systemic therapy for Stage IV non-small-cell lung cancer: American Society of Clinical Oncology clinical practice guideline update. J. Clin. Oncol..

[21] Travis WD (2011). International Association for the Study of Lung Cancer/American Thoracic Society/European Respiratory Society international multidisciplinary classification of lung adenocarcinoma. J. Thorac. Oncol..

[22] Travis WD (2015). WHO Classification of Tumours of the Lung, Pleura, Thymus and Heart.

[23] Hatabu H (2020). Interstitial lung abnormalities detected incidentally on CT: A position paper from the Fleischner society. Lancet Respir. Med..

[24] Podolanczuk AJ (2021). Update in interstitial lung disease 2020. Am. J. Respir. Crit. Care Med..

[25] Goldstraw P (2016). The IASLC lung cancer staging project: Proposals for revision of the TNM stage groupings in the forthcoming (Eighth) edition of the TNM classification for lung cancer. J. Thorac Oncol..

[26] Therasse P (2000). New guidelines to evaluate the response to treatment in solid tumors. European Organization for Research and Treatment of Cancer, National Cancer Institute of the United States, National Cancer Institute of Canada. J. Natl. Cancer Inst..

[27] Kanda Y (2013). Investigation of the freely available easy-to-use software “EZR” for medical statistics. Bone Marrow Transplant..

[28] Kasahara N (2017). Amrubicin monotherapy may be an effective second-line treatment for patients with large-cell neuroendocrine carcinoma or high-grade non-small-cell neuroendocrine carcinoma. Mol. Clin. Oncol..

[29] Yoshida H (2011). Amrubicin monotherapy for patients with previously treated advanced large-cell neuroendocrine carcinoma of the lung. Jpn. J. Clin. Oncol..

[30] Shimada Y (2012). Clinical features of unresectable high-grade lung neuroendocrine carcinoma diagnosed using biopsy specimens. Lung Cancer.

[31] Vallières E (2009). The IASLC Lung Cancer Staging Project: Proposals regarding the relevance of TNM in the pathologic staging of small cell lung cancer in the forthcoming (seventh) edition of the TNM classification for lung cancer. J. Thorac. Oncol..

[32] Tokito T (2014). Comparison of chemotherapeutic efficacy between LCNEC diagnosed using large specimens and possible LCNEC diagnosed using small biopsy specimens. Int. J. Clin. Oncol..

