# Supplementary figures and images for: Efficacy of second-line chemotherapy in patients with pulmonary large cell neuroendocrine carcinoma
Source: Sci Rep. 2024 Apr 1;14:7641. doi: 10.1038/s41598-024-58327-w (PMC10984918; doi:10.1038/s41598-024-58327-w)

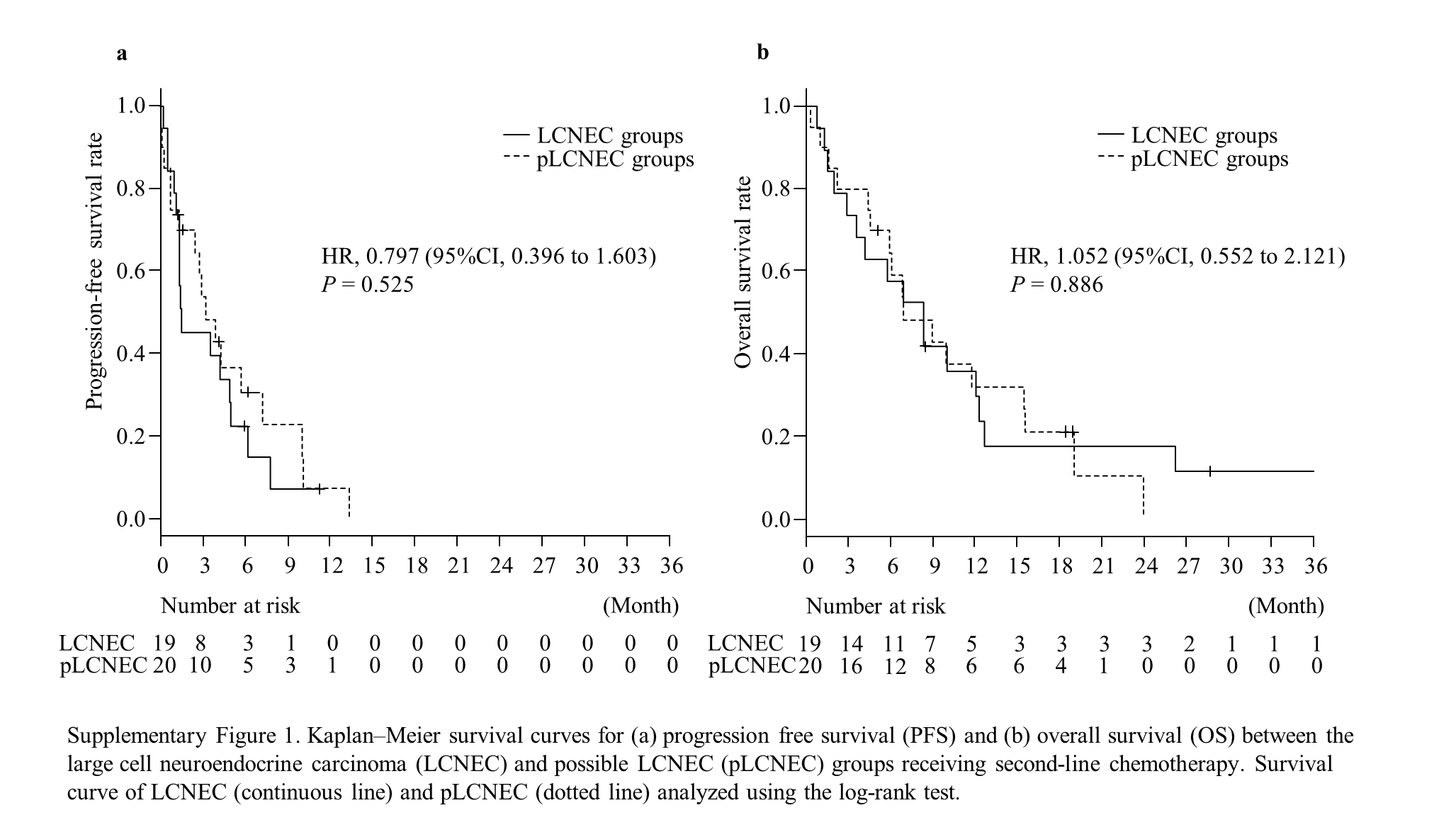

Supplement: Supplementary file 1 — Supplementary Figure S1. [file 41598_2024_58327_MOESM1_ESM.tif]
