# Supplementary material for: Efficacy of second-line chemotherapy in patients with pulmonary large cell neuroendocrine carcinoma
Source: Sci Rep. 2024 Apr 1;14:7641. doi: 10.1038/s41598-024-58327-w (PMC10984918; doi:10.1038/s41598-024-58327-w)
Supplement: Supplementary file 2 — Supplementary Table 1. [file 41598_2024_58327_MOESM2_ESM.docx]

**Supplementary Information**

Supplementary Table 1. Characteristics of the patients who were treated with amrubicin at the start of second-line chemotherapy.

|  | LCNEC | SCLC | P value |
| --- | --- | --- | --- |
| N | 24 | 208 |  |
| Median age (range) | 67 (58–83) | 68 (43–87) | 0.928 |
| Sex, n (%) |  |  |  |
| Female | 5 (20.8) | 43 (20.7) | 1.000 |
| Male | 19 (79.2) | 165 (79.3) |  |
| Smoking status, n (%) |  |  |  |
| Never | 1 (4.2) | 4 (1.9) | 0.424 |
| Ever smoker | 23 (95.8) | 204 (98.1) |  |
| ECOG performance status, n (%) |  |  |  |
| 0 | 7 (29.2) | 42 (20.2) | 0.740 |
| 1 | 15 (62.5) | 138 (66.3) |  |
| 2 | 2 (8.3) | 25 (12.0) |  |
| 3 | 0 (0.0) | 2 (1.0) |  |
| 4 | 0 (0.0) | 1 (0.5) |  |
| Stage at diagnosis  (8th TMN classification), n (%) |  |  |  |
| 1 | 4 (16.7) | 2 (1.0) | <0.001 |
| 2 | 2 (8.3) | 2 (1.0) |  |
| 3 | 3 (12.5) | 37 (17.8) |  |
| 4 | 15 (62.5) | 167 (80.3) |  |
| Treatment free survival, n (%) |  |  |  |
| > 90 days | 5 (20.8) | 47 (22.6) | 1.000 |
| ≤ 90 days | 19 (79.2) | 161 (77.4) |  |
| Histology of thoracic radiation therapy, n (%) |  |  |  |
| No | 21 (87.5) | 183 (88.0) | 1.000 |
| Yes | 3 (12.5) | 25 (12.0) |  |
| Histology of thoracic surgery, n (%) |  |  |  |
| No | 17 (70.8) | 199 (95.7) | <0.001 |
| Yes | 7 (29.2) | 9 (4.3) |  |
| Complication of interstitial lung disease, n (%) |  |  |  |
| No | 23 (95.8) | 192 (92.3) | 1.000 |
| Yes | 1 (4.2) | 16 (7.7) |  |

LCNEC: large cell neuroendocrine carcinoma, SCLC: small cell lung cancer, ILD: interstitial lung disease, ECOG: Eastern Cooperative Oncology Group.
